# Simultaneous assessment of stress hyperglycemia ratio and glycemic variability to predict mortality in patients with coronary artery disease: a retrospective cohort study from the MIMIC-IV database

**DOI:** 10.1186/s12933-024-02146-w

**Published:** 2024-02-09

**Authors:** Hao-ming He, Shu-wen Zheng, Ying-ying Xie, Zhe Wang, Si-qi Jiao, Fu-rong Yang, Xue-xi Li, Jie Li, Yi-hong Sun

**Affiliations:** 1 Department of Cardiology, China-Japan Friendship Hospital (Institute of Clinical Medical Sciences), Chinese Academy of Medical Sciences & Peking Union Medical College, Beijing, China; 2 Department of Cardiology, Beijing University of Chinese Medicine School of Traditional Chinese Medicine, Beijing, China; 3 Department of Cardiology, Peking University China-Japan Friendship School of Clinical Medicine, Beijing, China

**Keywords:** Coronary artery disease, Glycemic variability, Stress hyperglycemia ratio, MIMIC-IV database, Mortality

## Abstract

**Background:**

Stress hyperglycemia and glycemic variability (GV) can reflect dramatic increases and acute fluctuations in blood glucose, which are associated with adverse cardiovascular events. This study aimed to explore whether the combined assessment of the stress hyperglycemia ratio (SHR) and GV provides additional information for prognostic prediction in patients with coronary artery disease (CAD) hospitalized in the intensive care unit (ICU).

**Methods:**

Patients diagnosed with CAD from the Medical Information Mart for Intensive Care-IV database (version 2.2) between 2008 and 2019 were retrospectively included in the analysis. The primary endpoint was 1-year mortality, and the secondary endpoint was in-hospital mortality. Levels of SHR and GV were stratified into tertiles, with the highest tertile classified as high and the lower two tertiles classified as low. The associations of SHR, GV, and their combination with mortality were determined by logistic and Cox regression analyses.

**Results:**

A total of 2789 patients were included, with a mean age of 69.6 years, and 30.1% were female. Overall, 138 (4.9%) patients died in the hospital, and 404 (14.5%) patients died at 1 year. The combination of SHR and GV was superior to SHR (in-hospital mortality: 0.710 vs. 0.689, *p* = 0.012; 1-year mortality: 0.644 vs. 0.615, *p* = 0.007) and GV (in-hospital mortality: 0.710 vs. 0.632, *p* = 0.004; 1-year mortality: 0.644 vs. 0.603, *p* < 0.001) alone for predicting mortality in the receiver operating characteristic analysis. In addition, nondiabetic patients with high SHR levels and high GV were associated with the greatest risk of both in-hospital mortality (odds ratio [OR] = 10.831, 95% confidence interval [CI] 4.494–26.105) and 1-year mortality (hazard ratio [HR] = 5.830, 95% CI 3.175–10.702). However, in the diabetic population, the highest risk of in-hospital mortality (OR = 4.221, 95% CI 1.542–11.558) and 1-year mortality (HR = 2.013, 95% CI 1.224–3.311) was observed in patients with high SHR levels but low GV.

**Conclusions:**

The simultaneous evaluation of SHR and GV provides more information for risk stratification and prognostic prediction than SHR and GV alone, contributing to developing individualized strategies for glucose management in patients with CAD admitted to the ICU.

**Supplementary Information:**

The online version contains supplementary material available at 10.1186/s12933-024-02146-w.

## Introduction

Coronary artery disease (CAD) remains one of the leading causes of morbidity and mortality worldwide [1]. In addition, the prevalence of glycemic abnormality and diabetes is increasing rapidly, which contributes to poor cardiovascular outcomes in the CAD population [2, 3].

Excess activation of the sympathetic nervous system [4] and stress-induced insulin resistance [5, 6] are frequently observed in patients admitted to the intensive care unit (ICU). In these patients, circulating glucose-elevating hormone (catecholamine, steroid, and glucagon) levels increase, resulting in the occurrence of stress hyperglycemia [7]. The stress hyperglycemia ratio (SHR) is a well-known indicator of stress hyperglycemia and is regarded as a marker of the seriousness of the condition in critically ill patients [8, 9]. Several studies have demonstrated an association between SHR and poor prognosis in patients with CAD [3, 9]. However, it is calculated using only single glucose data on admission, which ignores the prognostic impact of glucose fluctuations during hospitalization. Hyperactivation of the sympathetic nervous system after acute stress also leads to impaired glucose homeostasis and drastic fluctuations in blood glucose [10]. Glycemic variability (GV) is usually used to indicate fluctuations in blood glucose and is associated with adverse cardiovascular events [11, 12].

Both stress hyperglycemia and GV are closely related to systemic inflammation, oxidative stress, and endothelial dysfunction, which contribute to poor prognosis [13–17]. Moreover, even transient exposure to stress hyperglycemia and acute high GV can also have long-term effects on cardiovascular events through inducing epigenetic modifications, which is called the “metabolic memory phenomenon” [18]. Therefore, the assessment of stress hyperglycemia and GV is of great importance for glycemic management and improvement in prognosis. However, no studies have explored the combination of SHR and GV to evaluate the prognosis in patients with CAD hospitalized in the ICU. In the present study, we assessed the prognostic value of SHR, GV, and their combination in CAD patients with or without diabetes who were admitted to the ICU.

## Methods

### Data source

For this study, we used data from the Medical Information Mart for Intensive Care-IV (MIMIC-IV) database (version 2.2) [19], which is a large publicly accessible data set containing > 50,000 deidentified records from ICU patients admitted to the Beth Israel Deaconess Medical Center (Boston, Massachusetts, USA) from 2008 to 2019 [20]. After the completion of the Collaborative Institutional Training Initiative program, one author (HM He) obtained access to the MIMIC-IV database (certification number: 55642259). This study was in accordance with the Declaration of Helsinki, and the requirement for informed consent was waived as anonymous data were analyzed. The institutional review board of Beth Israel Deaconess Medical Center (Boston, Massachusetts, USA) approved the study.

### Study population

**Fig. 1:**
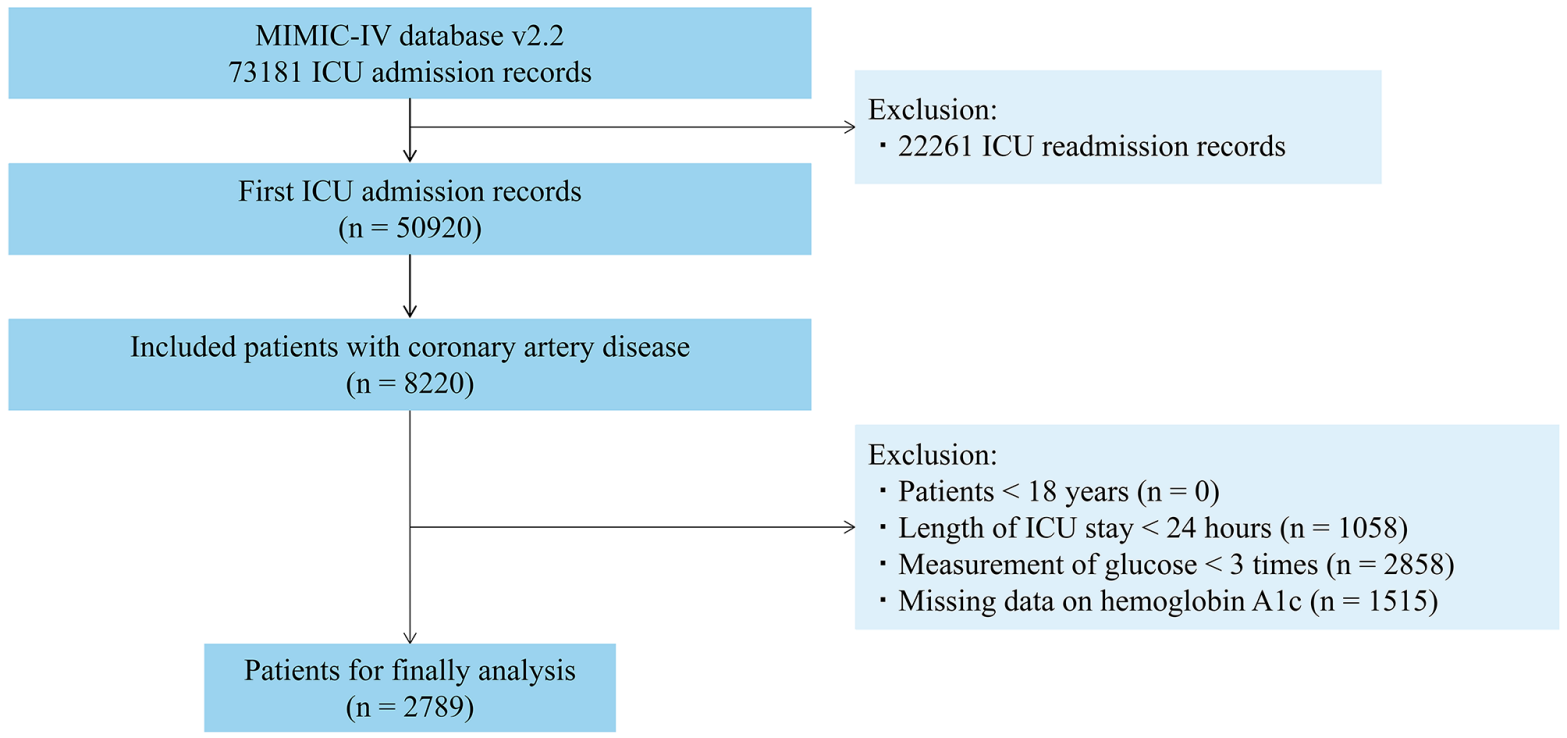
Flowchart of patient inclusion and exclusion from the MIMIC-IV database. Abbreviations: HbA1c, glycated hemoglobin A1c; ICU, intensive care unit; MIMIC-IV, Medical Information Mart for Intensive Care-IV

From the MIMIC-IV database, ICU patients with a diagnosis of CAD (the top 3 ranks) according to the International Classification of Diseases (ICD)-9 codes (410.xx-414.xx) and the ICD-10 codes (I20.x-I22.x, and I25.x) were retrospectively included in our analysis. For patients with multiple ICU hospitalizations, only the first hospitalization record was considered in our analysis. We excluded patients who met the following criteria: less than 18 years (*n* = 0), length of ICU stay < 24 h (*n* = 1058), measurement of blood glucose < 3 times (*n* = 2858), and lack data on hemoglobin A1c (HbA1c) (*n* = 1515). A total of 2789 patients were included in the final cohort. The flowchart of the patient inclusion process is shown in Fig. 1.

### Data extraction and definitions

The extraction of demographic characteristics, vital signs, comorbidities, treatment during ICU stay, and laboratory measurements were performed using Navicat Premium software (version 15.0.29) with a structured query language. According to the ICD-9 codes 250.0x-250.9x and ICD-10 codes E10-E14, diabetes was ascertained. Codes for data extraction from the MIMIC-IV database can be downloaded from https://github.com/MIT-LCP/mimic-code, which were provided by the Massachusetts Institute of Technology Laboratory for Computational Physiology. Two investigators (HM He and SW Zheng) will check for reliability after the relevant data extraction. For vital signs and laboratory measurements, data from the first 24 h of ICU admission were extracted.

SHR was calculated by the following equation: [plasma glucose (mg/dL)/(28.7 × HbA1c (%) − 46.7)] [8]. GV was expressed as the coefficient of variation, defined as the ratio of the standard deviation to the mean of all repeat plasma glucose measurements during the ICU stay [12, 21]. Each patient had blood glucose measured at least three times during their ICU stay. The timing of blood glucose measurement was determined by the physician. The estimated glomerular filtration rate (eGFR) was calculated using the modified Modification of Diet in Renal Disease formula [22]. Antidiabetic agents include insulin and other antidiabetic drugs (including metformin, sulfonylureas, thiazolidinediones, dipeptidyl peptidase-4 inhibitors, alpha-glucosidase inhibitors, glucagon-like peptide-1 [GLP-1] receptor agonists, and sodium-glucose cotransporter-2 [SGLT-2] inhibitors).

### Study endpoint

The primary endpoint of the present study was 1-year mortality, and the secondary endpoint was in-hospital mortality. Death information was derived from either state or hospital death records. All patients in the MIMIC-IV database were followed up for at least one year.

### Statistical analysis

Data are presented as the means ± standard deviations for continuous normally distributed variables, medians (interquartile ranges) for skewed continuous variables, and frequencies (percentages) for categorical variables. For between-group comparisons, Student’s t test, Mann-Whitney U test, chi-squared test, and Fisher’s exact test were performed, as appropriate. We divided the distribution of SHR and GV into tertiles (SHR: <0.89, 0.89–1.16, > 1.16; GV: <15.7, 15.7–27.3, > 27.3), with the highest tertile classified as high and the lower two tertiles classified as low. Receiver operating characteristic curves and Harrell’s C-statistic were used to assess the prognostic value of SHR, GV, and their combination for in-hospital and 1-year mortality. Kaplan-Meier curves were plotted, and log-rank statistics were calculated for survival analyses. Logistic regression analysis and Cox proportional hazard regression analysis were conducted to investigate the associations of SHR, GV, and their combination with mortality. Univariate analyses were performed first for each potential factor, and both significant variables (*p* < 0.05) in univariate analysis and variables that were considered clinically relevant were included in multivariate analyses. For the multivariate logistic regression analysis, we adjusted for age, female, body mass index, heart rate, acute myocardial infarction (AMI), chronic heart failure, peripheral vascular disease, cerebrovascular disease, angiotensin-converting enzyme inhibitors/angiotensin receptor blockers (ACEIs/ARBs), vasoactive drugs, insulin, other antidiabetic drugs, renal replacement therapy, ventilation, and eGFR. For the multivariate Cox regression analysis, we adjusted for age, female, body mass index, diastolic blood pressure, heart rate, AMI, hypertension, diabetes, chronic heart failure, peripheral vascular disease, cerebrovascular disease, antiplatelets, ACEIs/ARBs, insulin, other antidiabetic drugs, renal replacement therapy, ventilation, and eGFR. In the sensitivity analysis, a forward stepwise selection algorithm (*p* < 0.05 to enter, *p* > 0.10 to exit) was used to construct models, and a backward stepwise variable selection procedure was used to confirm the models. Restricted cubic spline curves with four knots were plotted to explore the potential nonlinear associations of SHR and GV with mortality. To graphically illustrate the results of subgroup analyses and to identify the potential interaction effects, forest plots were used. All analyses were performed with R statistical software (R Foundation for Statistical Computing, Vienna, Austria, version 4.3.0) and SPSS statistics software (version 25.0, SPSS, Inc., Chicago, Illinois, United States). The value of two-tailed *p* < 0.05 was considered to be statistically significant.

## Results

### Baseline characteristics

We identified a total of 2789 patients eligible for the analysis. The baseline characteristics of the study population are shown in Table 1. Overall, the mean age was 69.6 ± 11.6 years, and 839 (30.1%) were female. Of these patients, 1480 (53.1%) had AMI, and 1326 (47.5%) had diabetes. Compared with survivors, nonsurvivors tended to be older and have a higher proportion of AMI, hypertension, diabetes, chronic heart failure, peripheral vascular disease, cerebrovascular disease, and chronic kidney disease. Nonsurvivors were less likely to receive antiplatelets, ACEIs/ARBs, other antidiabetic drugs, and ventilation but more likely to receive renal replacement therapy than survivors. For patients who died, there were higher levels of serum creatinine, white blood cells, platelets, glucose, SHR, and GV but lower levels of eGFR and hemoglobin.

**Table 1:** Baseline characteristics of study population

Variables	Survivors (*n* = 2385)	Non-survivors (*n* = 404)	*p*-value
**Demographics**			
Age, years	68.8 ± 11.4	74.5 ± 11.8	< 0.001
Age ≥ 75 years, *n* (%)	741 (31.1)	216 (53.5)	< 0.001
Female, *n* (%)	680 (28.5)	159 (39.4)	< 0.001
Body mass index, kg/m^2^	29.5 ± 5.9	28.8 ± 7.2	0.115
**Vital signs**			
Systolic blood pressure, mmHg	118.8 ± 21.6	120.8 ± 25.2	0.124
Diastolic blood pressure, mmHg	63.7 ± 15.5	66.7 ± 19.6	0.003
Heart rate, bpm	82.3 ± 14.4	87.6 ± 19.7	< 0.001
**Comorbidities**			
AMI, *n* (%)	1176 (49.3)	304 (75.2)	< 0.001
Hypertension, *n* (%)	1908 (80.0)	348 (86.1)	0.005
Diabetes, *n* (%)	1112 (46.6)	214 (53.0)	0.021
History of MI, *n* (%)	399 (16.7)	61 (15.1)	0.457
Chronic heart failure, *n* (%)	887 (37.2)	262 (64.9)	< 0.001
Peripheral vascular disease, *n* (%)	368 (15.4)	88 (21.8)	0.002
Cerebrovascular disease, *n* (%)	329 (13.8)	113 (28.0)	< 0.001
Chronic kidney disease, *n* (%)	722 (30.3)	263 (65.1)	< 0.001
**Treatment during hospitalization**			
Antiplatelets, *n* (%)	2055 (86.2)	331 (82.1)	0.038
Statins, *n* (%)	1838 (77.1)	298 (73.9)	0.187
ACEIs/ARBs, *n* (%)	648 (27.2)	90 (22.3)	0.048
Beta-blockers, *n* (%)	1240 (52.0)	216 (53.6)	0.593
Vasoactive drugs, *n* (%)	1572 (65.9)	258 (63.9)	0.456
Insulin, *n* (%)	1405 (58.9)	230 (56.9)	0.489
Other antidiabetic drugs, *n* (%)	160 (6.7)	12 (3.0)	0.005
Renal replacement therapy, *n* (%)	103 (4.3)	79 (19.6)	< 0.001
Ventilation, *n* (%)	1885 (79.0)	288 (71.3)	0.001
**Laboratory measurements**			
Serum creatinine, mg/dL	1.0 (0.8–1.3)	1.4 (1.0–2.0)	< 0.001
eGFR, mL/min/1.73m^2^	76.3 (55.9–96.4)	48.4 (32.5–68.5)	< 0.001
White blood cells, 10^9^/L	10.8 (7.9–14.6)	11.7 (8.6–15.8)	0.005
Hemoglobin, g/dL	11.0 ± 2.5	10.7 ± 2.3	0.034
Platelets, 10^9^/L	196.4 ± 82.4	223.1 ± 102.3	< 0.001
Glucose, mg/dL	137.0 (115.0-178.0)	178.0 (133.5–260.0)	< 0.001
HbA1c, %	6.6 ± 1.6	6.6 ± 1.5	0.453
SHR, (mean ± standard deviation)	1.1 ± 0.5	1.3 ± 0.6	< 0.001
GV, %	20.0 (12.9–30.6)	25.2 (16.9–36.7)	< 0.001
Length of ICU stay, days	3.0 (2.0–4.0)	4.0 (2.0–7.0)	< 0.001
SOFA score, (mean ± standard deviation)	5.0 (3.0–7.0)	6.0 (4.0–8.0)	< 0.001

Abbreviations: ACEIs/ARBs, angiotensin-converting enzyme inhibitors/angiotensin receptor blockers; AMI, acute myocardial infarction; eGFR, estimated glomerular filtration rate; GV, glycemic variability; HbA1c, glycated hemoglobin A1c; ICU, intensive care unit; MI, myocardial infarction; SHR, stress hyperglycemia ratio; SOFA, Sequential Organ Failure Assessment

### The association between SHR and mortality in patients with or without diabetes

**Fig. 2:**
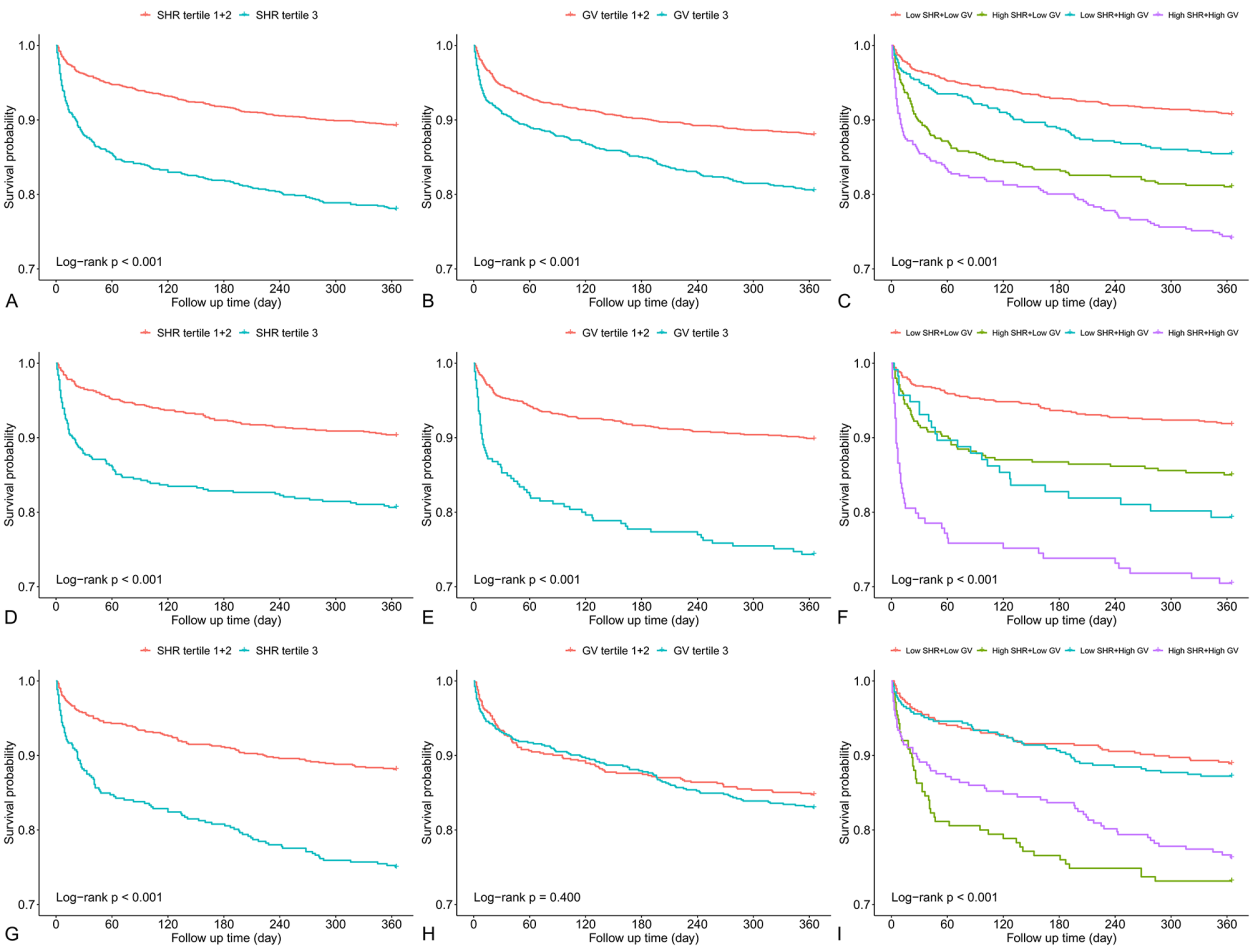
Kaplan-Meier curves of SHR, GV, and their combination for 1-year mortality. **(A-C)** Overall population; **(D-F)** patients without diabetes; **(G-I)** patients with diabetes. Abbreviations: GV, glycemic variability; SHR, stress hyperglycemia ratio

There were 138 (4.9%) patients who died during their hospital stay, and 404 (14.5%) patients died during their 1-year follow-up period. Kaplan-Meier curves of SHR for 1-year mortality are presented in Fig. 2A and D, and 2G. The results of the univariate analysis are shown in Supplementary Table 1.

**Fig. 3:**
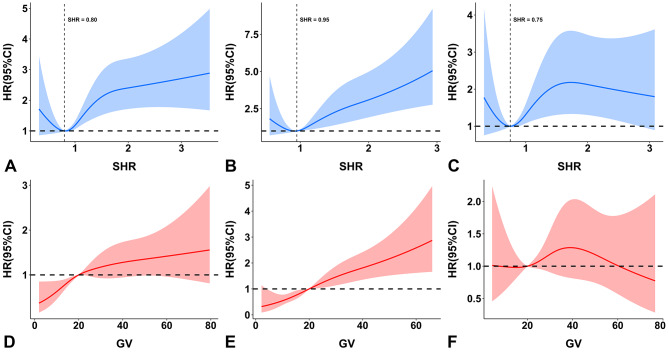
Multivariable-adjusted restricted cubic spline analyses of SHR and GV for 1-year mortality. Adjusted for covariates as in Table 2. (**A** and **D**) Overall population; (**B** and **E**) patients without diabetes; (**C** and **F**) patients with diabetes. Abbreviations: CI, confidence interval; GV, glycemic variability; HR, hazard ratio; SHR, stress hyperglycemia ratio

**Fig. 4:**
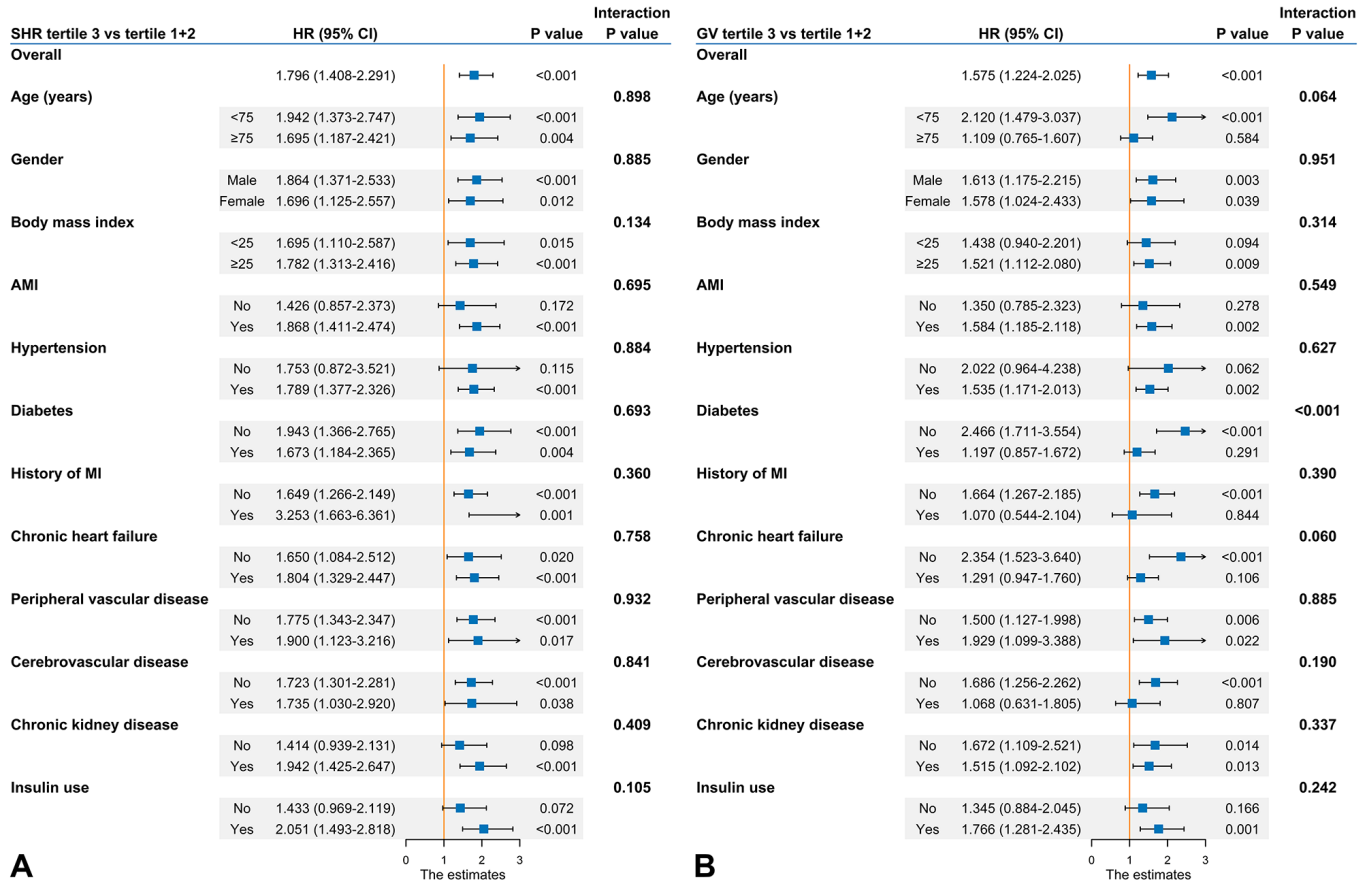
Forest plots for subgroup analyses of **(A)** SHR and **(B)** GV with 1-year mortality. Abbreviations: AMI, acute myocardial infarction; CI, confidence interval; HR, hazard ratio; MI, myocardial infarction

In the overall population, SHR was an independent risk factor for in-hospital mortality (odds ratio [OR] = 2.149 per 1-point increment, 95% confidence interval [CI] 1.557–2.967) and 1-year mortality (hazard ratio [HR] = 1.470 per 1-point increment, 95% CI 1.239–1.745) after adjustment. In addition, patients in the highest tertile group had a 3.9-fold or a 1.7-fold higher risk of in-hospital mortality and 1-year mortality than those in the lowest tertile group, respectively. Similar associations were observed in the nondiabetic and diabetic populations (Table 2). There was a U-shaped relationship between SHR and 1-year mortality, with SHR 0.80, 0.95, and 0.75 having the lowest risk in the overall population, nondiabetic population, and diabetic population, respectively (Fig. 3A-C). The interaction effects were not significant in the subgroup analysis, indicating the robustness of the results (Fig. 4).

**Table 2:** The associations of SHR and GV with mortality in patients with or without diabetes

	Continuous	Categorical		
	Per 1-unit increment	Tertile 1	Tertile 2	Tertile 3
**In-hospital mortality**				
**Overall**				
SHR				
Unadjusted	2.288 (1.835–2.854)^‡^	1.000	0.849 (0.483–1.492)	3.508 (2.256–5.453)^‡^
Model 1	3.023 (2.314–3.950)^‡^	1.000	1.086 (0.551–2.143)	5.564 (3.246–9.537)^‡^
Model 2	2.149 (1.557–2.967)^‡^	1.000	1.011 (0.493–2.071)	3.948 (2.185–7.136)^‡^
GV				
Unadjusted	1.021 (1.012–1.031)^‡^	1.000	2.531 (1.492–4.292)^‡^	3.651 (2.200−6.057)^‡^
Model 1	1.023 (1.012–1.034)^‡^	1.000	2.736 (1.473–5.083)^†^	3.963 (2.176–7.218)^‡^
Model 2	1.019 (1.006–1.032)^‡^	1.000	1.671 (0.860–3.248)	2.468 (1.288–4.728)^†^
**Patients without diabetes**				
SHR				
Unadjusted	4.556 (3.161–6.568)^‡^	1.000	0.536 (0.225–1.276)	3.818 (1.965–7.418)^‡^
Model 1	5.111 (3.406–7.670)^‡^	1.000	0.516 (0.190–1.404)	4.802 (2.302–10.015)^‡^
Model 2	3.063 (1.931–4.860)^‡^	1.000	0.414 (0.141–1.212)	2.992 (1.316–6.804)^†^
GV				
Unadjusted	1.044 (1.029–1.058)^‡^	1.000	4.516 (2.119–9.628)^‡^	11.825 (5.610−24.925)^‡^
Model 1	1.043 (1.028–1.059)^‡^	1.000	5.213 (2.100-12.936)^‡^	15.104 (6.192–36.842)^‡^
Model 2	1.039 (1.019–1.059)^‡^	1.000	3.770 (1.413–10.057)^†^	8.714 (3.262–23.283)^‡^
**Patients with diabetes**				
SHR				
Unadjusted	1.544 (1.146–2.081)^†^	1.000	1.440 (0.684–3.033)	3.047 (1.663–5.582)^‡^
Model 1	1.992 (1.361–2.917)^‡^	1.000	2.344 (0.913–6.021)	6.031 (2.694–13.498)^‡^
Model 2	1.603 (0.991–2.595)	1.000	1.993 (0.703–5.650)	5.334 (2.151–13.227)^‡^
GV				
Unadjusted	1.001 (0.985–1.017)	1.000	1.064 (0.501–2.261)	1.108 (0.551–2.228)
Model 1	0.998 (0.978–1.019)	1.000	1.042 (0.437–2.482)	0.946 (0.411–2.175)
Model 2	0.996 (0.972–1.021)	1.000	0.657 (0.236–1.828)	0.796 (0.303–2.096)
**1-year mortality**				
**Overall**				
SHR				
Unadjusted	1.639 (1.457–1.845)^‡^	1.000	0.941 (0.713–1.242)	2.155 (1.700−2.731)^‡^
Model 1	1.972 (1.716–2.265)^‡^	1.000	1.084 (0.785–1.498)	2.683 (2.031–3.544)^‡^
Model 3	1.470 (1.239–1.745)^‡^	1.000	0.928 (0.665–1.295)	1.726 (1.279–2.330)^‡^
GV				
Unadjusted	1.017 (1.012–1.023)^‡^	1.000	1.687 (1.287–2.210)^‡^	2.271 (1.755–2.939)^‡^
Model 1	1.019 (1.012–1.025)^‡^	1.000	1.710 (1.245–2.348)^‡^	2.396 (1.764–3.253)^‡^
Model 3	1.014 (1.007–1.022)^‡^	1.000	1.450 (1.046–2.010)^*^	1.991 (1.427–2.777)^‡^
**Patients without diabetes**				
SHR				
Unadjusted	2.273 (1.835–2.815)^‡^	1.000	0.799 (0.531–1.203)	1.882 (1.301–2.721)^‡^
Model 1	2.718 (2.174–3.397)^‡^	1.000	0.968 (0.596–1.569)	2.617 (1.705–4.018)^‡^
Model 3	2.070 (1.572–2.725)^‡^	1.000	0.762 (0.464–1.251)	1.644 (1.039–2.599)^*^
GV				
Unadjusted	1.030 (1.023–1.038)^‡^	1.000	2.096 (1.460–3.011)^‡^	4.089 (2.832–5.904)^‡^
Model 1	1.030 (1.023–1.038)^‡^	1.000	2.161 (1.408–3.318)^‡^	4.615 (2.998–7.105)^‡^
Model 3	1.028 (1.019–1.038)^‡^	1.000	1.824 (1.173–2.836)^†^	3.459 (2.190–5.463)^‡^
**Patients with diabetes**				
SHR				
Unadjusted	1.445 (1.244–1.678)^‡^	1.000	1.213 (0.823–1.787)	2.476 (1.814–3.378)^‡^
Model 1	1.680 (1.393–2.026)^‡^	1.000	1.390 (0.890–2.171)	2.926 (2.017–4.246)^‡^
Model 3	1.244 (0.975–1.588)	1.000	1.052 (0.665–1.666)	1.714 (1.138–2.582)^†^
GV				
Unadjusted	1.005 (0.996–1.013)	1.000	1.056 (0.703–1.587)	1.161 (0.798–1.689)
Model 1	1.002 (0.991–1.013)	1.000	0.990 (0.616–1.591)	1.073 (0.688–1.673)
Model 3	1.003 (0.991–1.014)	1.000	0.967 (0.597–1.566)	1.171 (0.739–1.855)

Model 1: adjusted for age, female, and body mass index

Model 2: adjusted for Model 1 plus heart rate, acute myocardial infarction, chronic heart failure, peripheral vascular disease, cerebrovascular disease, angiotensin-converting enzyme inhibitors/angiotensin receptor blockers, vasoactive drugs, insulin, other antidiabetic drugs, renal replacement therapy, ventilation, and estimated glomerular filtration rate

Model 3: adjusted for Model 1 plus diastolic blood pressure, heart rate, acute myocardial infarction, hypertension, diabetes, chronic heart failure, peripheral vascular disease, cerebrovascular disease, antiplatelets, angiotensin-converting enzyme inhibitors/angiotensin receptor blockers, insulin, other antidiabetic drugs, renal replacement therapy, ventilation, and estimated glomerular filtration rate

^*^P < 0.05, ^†^*P* < 0.01, ^‡^*P* < 0.001

### The association between GV and mortality in patients with or without diabetes

Kaplan-Meier curves of GV for 1-year mortality are presented in Fig. 2B and E, and 2H. In the logistic and Cox regression analyses, GV was independently associated with mortality in the overall population and nondiabetic population. However, no significant associations of GV with in-hospital and 1-year mortality were observed in the diabetic population regardless of whether GV was treated as a continuous variable (in-hospital mortality: OR = 0.996 per 1-percent increment, 95% CI 0.972–1.021; 1-year mortality: HR = 1.003 per 1-percent increment, 95% CI 0.991–1.014) or a categorical variable (in-hospital mortality: highest tertile vs. lowest tertile: OR = 0.796, 95% CI 0.303–2.096; 1-year mortality: highest tertile vs. lowest tertile: HR = 1.171, 95% CI 0.739–1.855) (Table 2). There were significant interaction effects between GV and diabetes for in-hospital (*p* for interaction < 0.001) (data not shown) and 1-year mortality (*p* for interaction < 0.001) (Fig. 4). Restricted cubic spline curves showed a clear linear association between GV and 1-year mortality in patients without diabetes (*p* nonlinearity = 0.148), but no obvious dose-response relationship was observed in patients with diabetes (Fig. 3D-F).

### The association of the combination of SHR and GV with mortality in patients with or without diabetes

**Fig. 5:**
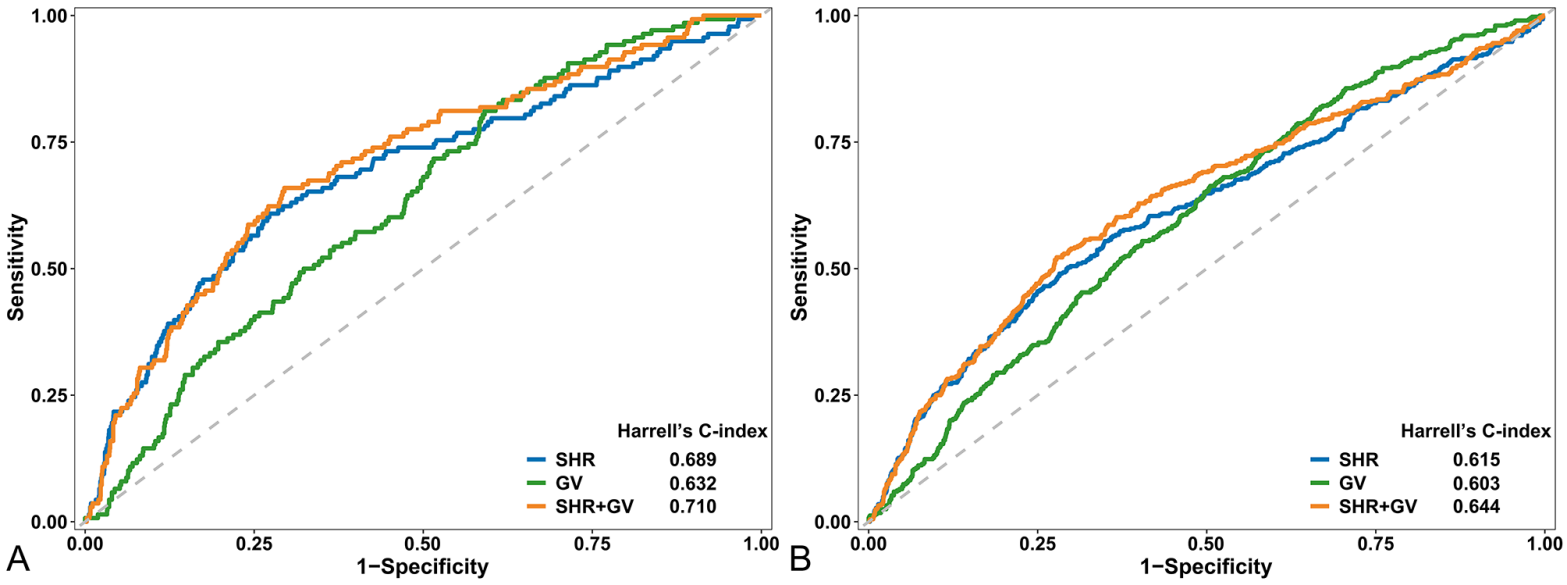
Receiver operating characteristic curves of SHR, GV, and their combination in predicting in-hospital and 1-year mortality. **(A)** In-hospital mortality; **(B)** 1-year mortality. Abbreviations: GV, glycemic variability; SHR, stress hyperglycemia ratio

Receiver operating characteristic curves of SHR, GV, and their combination are plotted in Fig. 5. The combination of SHR and GV performed better than SHR (in-hospital mortality: 0.710 vs. 0.689, *p* = 0.012; 1-year mortality: 0.644 vs. 0.615, *p* = 0.007) and GV (in-hospital mortality: 0.710 vs. 0.632, *p* = 0.004; 1-year mortality: 0.644 vs. 0.603, *p* < 0.001) alone in predicting mortality.

Kaplan-Meier curves of the combination of SHR and GV for 1-year mortality are presented in Fig. 2C and F, and 2I. In the nondiabetic population, patients with high SHR levels and high GV (SHR > 1.16 and GV > 27.3) had the greatest risk of both in-hospital mortality (OR = 10.831, 95% CI 4.494–26.105) and 1-year mortality (HR = 5.830, 95% CI 3.175–10.702). However, patients with high SHR levels but low GV (SHR > 1.16 and GV < 27.3) showed the highest in-hospital mortality (OR = 4.221, 95% CI 1.542–11.558) and 1-year mortality (HR = 2.013, 95% CI 1.224–3.311) in the diabetic population (Table 3).

**Table 3:** The association of the combination of SHR and GV with mortality in patients with or without diabetes

	Group			
	Low SHR and low GV (SHR < 1.16 and GV < 27.3)	High SHR and low GV (SHR > 1.16 and GV < 27.3)	Low SHR and high GV (SHR < 1.16 and GV > 27.3)	High SHR and high GV (SHR > 1.16 and GV > 27.3)
**In-hospital mortality**				
**Overall**				
Unadjusted	1.000	3.520 (2.163–5.731)^‡^	1.734 (0.975–3.081)	5.984 (3.743–9.567)^‡^
Model 1	1.000	4.664 (2.656–8.190)^‡^	1.553 (0.770–3.131)	8.416 (4.862–14.568)^‡^
Model 2	1.000	3.694 (1.979–6.898)^‡^	1.447 (0.685–3.056)	5.436 (2.921–10.118)^‡^
Model 4	1.000	3.582 (1.917–6.694)^‡^	1.407 (0.667–2.967)	5.163 (2.768–9.630)^‡^
**Patients without diabetes**				
Unadjusted	1.000	3.956 (2.039–7.678)^‡^	3.040 (1.155–7.999)^*^	14.050 (7.343–26.884)^‡^
Model 1	1.000	5.508 (2.524–12.024)^‡^	4.098 (1.447–11.604)^†^	20.024 (9.313–43.052)^‡^
Model 2	1.000	4.347 (1.823–10.369)^‡^	3.294 (1.057–10.271)^*^	10.831 (4.494–26.105)^‡^
Model 4	1.000	4.387 (1.836–10.482)^‡^	3.280 (1.046–10.282)^*^	11.302 (4.593–27.813)^‡^
**Patients with diabetes**				
Unadjusted	1.000	3.166 (1.531–6.551)^†^	1.121 (0.535–2.351)	2.512 (1.254–5.032)^†^
Model 1	1.000	4.111 (1.791–9.433)^‡^	0.757 (0.290–1.979)	3.307 (1.463–7.475)^†^
Model 2	1.000	4.221 (1.542–11.558)^†^	0.988 (0.343–2.845)	3.664 (1.365–9.830)^†^
Model 4	1.000	4.234 (1.522–11.779)^†^	0.913 (0.314–2.653)	3.714 (1.364–10.113)^*^
**1-year mortality** ^**a**^				
**Overall**				
Unadjusted	1.000	2.243 (1.702–2.957)^‡^	1.680 (1.215–2.324)^†^	3.336 (2.372–4.692)^‡^
Model 1	1.000	3.024 (2.184–4.187)^‡^	2.137 (1.454–3.140)^‡^	4.667 (3.088–7.051)^‡^
Model 3	1.000	2.121 (1.512–2.975)^‡^	1.980 (1.322–2.966)^‡^	2.975 (1.932–4.579)^‡^
Model 5	1.000	2.029 (1.443–2.852)^‡^	1.887 (1.259–2.829)^†^	2.861 (1.859–4.402)^‡^
**Patients without diabetes**				
Unadjusted	1.000	2.193 (1.492–3.222)^‡^	3.404 (2.025–5.722)^‡^	5.998 (3.704–9.714)^‡^
Model 1	1.000	3.272 (2.066–5.183)^‡^	4.770 (2.614–8.705)^‡^	9.336 (5.248–16.605)^‡^
Model 3	1.000	2.409 (1.492–3.891)^‡^	3.975 (2.129–7.422)^‡^	5.830 (3.175–10.702)^‡^
Model 5	1.000	2.243 (1.381–3.641)^†^	3.951 (2.124–7.351)^‡^	5.169 (2.806–9.521)^‡^
**Patients with diabetes**				
Unadjusted	1.000	2.589 (1.727–3.880)^‡^	1.059 (0.685–1.636)	2.003 (1.223–3.279)^†^
Model 1	1.000	3.170 (1.975–5.088)^‡^	1.158 (0.688–1.949)	2.391 (1.304–4.382)^†^
Model 3	1.000	2.013 (1.224–3.311)^†^	1.343 (0.787–2.294)	1.804 (0.962–3.382)
Model 5	1.000	2.006 (1.218–3.303)^†^	1.309 (0.765–2.239)	1.837 (0.978–3.451)

Abbreviations: GV, glycemic variability; SHR, stress hyperglycemia ratio

^a^The assumption of proportional hazards was not met. Therefore, HRs were calculated using Cox regression analysis with time-dependent covariates

Model 1: adjusted for age, female, and body mass index

Model 2: adjusted for Model 1 plus heart rate, acute myocardial infarction, chronic heart failure, peripheral vascular disease, cerebrovascular disease, angiotensin-converting enzyme inhibitors/angiotensin receptor blockers, vasoactive drugs, insulin, other antidiabetic drugs, renal replacement therapy, ventilation, and estimated glomerular filtration rate

Model 3: adjusted for Model 1 plus diastolic blood pressure, heart rate, acute myocardial infarction, hypertension, diabetes, chronic heart failure, peripheral vascular disease, cerebrovascular disease, antiplatelets, angiotensin-converting enzyme inhibitors/angiotensin receptor blockers, insulin, other antidiabetic drugs, renal replacement therapy, ventilation, and estimated glomerular filtration rate

Model 4 (sensitivity analysis): adjusted for Model 2 plus the Sequential Organ Failure Assessment score

Model 5 (sensitivity analysis): adjusted for Model 3 plus the Sequential Organ Failure Assessment score

^*^P < 0.05, ^†^*P* < 0.01, ^‡^*P* < 0.001

### Sensitivity analysis

We performed a sensitivity analysis where a forward stepwise selection algorithm (*p* < 0.05 to enter, *p* > 0.10 to exit) was used to construct models, and a backward stepwise selection procedure was used to confirm the models. We still found that patients with high SHR levels and high GV (SHR > 1.16 and GV > 27.3) showed the highest risk of mortality in the nondiabetic population. However, in the diabetic population, patients with high SHR levels but low GV (SHR > 1.16 and GV < 27.3) showed the greatest risk of mortality (Supplementary Table 2).

Several previous studies have used blood glucose measurements within 72 h after admission to calculate GV [23–25]. Furthermore, the median ICU length of stay was 3 days (interquartile range: 2–4 days) in our study. Therefore, we also performed a sensitivity analysis that only used blood glucose measurements within 72 h after ICU admission to calculate GV. We found that our main results remained unchanged, suggesting the robustness of our results (Supplementary Table 3).

Previous studies have reported that GV was associated with the seriousness of the condition in critically ill patients [26, 27]. Therefore, we conducted a sensitivity analysis that further adjusted for the Sequential Organ Failure Assessment (SOFA) score, which was an indicator of the disease severity. We found that our main results remained unchanged (results of model 4 and model 5 in Table 3).

## Discussion

In the present study, we evaluated the effect of combining SHR and GV on mortality in patients with CAD hospitalized in the ICU. Several novel findings of our study have clinical implications and deserve emphasis. (1) A U-shaped relationship between SHR and 1-year mortality was observed regardless of diabetes status. (2) GV was significantly associated with mortality in patients without diabetes. However, it was not independently associated with mortality after adjustment in patients with diabetes. (3) In the nondiabetic population, patients with high levels of both SHR and GV have the poorest prognosis. However, it is worth noting that patients with high SHR levels but low GV showed the worst prognosis in the diabetic population. Therefore, simultaneous assessment of SHR and GV can provide better risk stratification and prognostic prediction in patients with CAD hospitalized in the ICU, which could compensate for the deficiency of SHR and GV alone in evaluating the risk of mortality.

Patients hospitalized in the ICU are at a higher risk of stress hyperglycemia and drastic fluctuations in blood glucose under the combined action of sympathetic nervous system hyperactivity [7, 10] and stress-induced insulin resistance [5, 6]. Evidence suggests that hyperglycemia can trigger oxidative stress and inflammatory responses, leading to myocardial injury and increased infarct size, which increases the risk of death [13, 17, 28, 29]. Furthermore, patients with hyperglycemia also show impaired nitric oxide bioavailability, an enhanced coagulation system, and reduced fibrinolytic activity, which promotes a prothrombotic environment and contributes to mortality [14, 15]. A prospective cohort study of patients with AMI indicated that admission hyperglycemia was significantly associated with adverse short- and long-term outcomes regardless of diabetes status [30]. SHR is a good indicator of stress hyperglycemia. The association between SHR and adverse cardiovascular events has been well-established in critically ill patients and patients with CAD [3, 9]. However, glucose-lowering treatments during hospitalization can affect blood glucose levels and subsequently influence the prognosis. The SHR calculated with the first admission glucose value cannot reflect the effect of acute glucose fluctuations during hospitalization on prognosis.

High GV is also a manifestation of impaired glucose homeostasis. It promotes increased coronary plaque vulnerability through similar mechanisms as for hyperglycemia, including inflammation and oxidative stress [31]. In addition, it also facilitates cardiac fibrosis [32] and adverse left ventricular remodeling [33], resulting in adverse cardiovascular events. Moreover, patients with high GV have an increased risk of hypoglycemia [34, 35], which can lead to myocardial ischemia, arrhythmias, and other complications through the activation of the sympathoadrenal system [36, 37]. Su et al. [21] retrospectively included 17,756 ICU patients from the MIMIC-IV database and indicated that high GV was independently associated with in-hospital mortality. Furthermore, the effect of GV on in-hospital mortality was partially mediated by ventricular arrhythmias. Patients with high GV have an increased risk of hypoglycemia and hyperglycemia events [38], linked to the activation of the sympathoadrenal system, which may increase the risk of ventricular arrhythmias [10, 37]. Gerbaud et al. [11] revealed that higher GV assessed by the standard deviation of glucose during hospitalization was significantly associated with midterm major adverse cardiac events in critically ill patients with diabetes and acute coronary syndrome. In addition, a prospective cohort study based on an acute heart failure population also confirmed the association between acute GV (defined by the coefficient of variation of glucose during hospitalization) and 1-year mortality in patients without diabetes, whereas this was not the case for diabetic patients [12]. In the present study, we found that GV was significantly associated with mortality in patients without diabetes, whereas this association was not observed in patients with diabetes. This finding was consistent with Chun et al. [12] and Su et al. [21]. This may be because patients with diabetes were already adapted to glucose fluctuations during their illness [39, 40]. In addition, compared with patients without diabetes, the cutoff values for detrimental low or high glucose are lower or higher in patients with diabetes, respectively [39, 41]. Therefore, patients with diabetes can tolerate a broader range of blood glucose than patients without diabetes.

Moreover, stress hyperglycemia and high GV promote the generation of mitochondrial reactive oxygen species and advanced glycation end products, which induce the process of epigenetic modifications (such as DNA methylation and posttranslational modification of histone proteins) [18]. These epigenetic modifications alter the expression of genes, and even short-term exposure to stress hyperglycemia and high GV can have a long-lasting impact on long-term cardiovascular outcomes, which is known as the “metabolic memory phenomenon” [18]. Many clinical studies and animal studies have demonstrated the relationship between hyperglycemia-induced metabolic memory phenomenon and cardiovascular disease [42, 43].

Given the importance of stress hyperglycemia and acute glucose fluctuations in prognostic prediction, the combination of SHR and GV may help to improve the risk stratification of severely ill patients. In a cohort of ICU patients diagnosed with sepsis, patients with severe hyperglycemia and low GV showed a higher risk of mortality, irrespective of whether they had diabetes [44]. The reason for the discrepancy between their study and our study may be attributed to different study populations and different metrics (i.e. mean blood glucose, not stress hyperglycemia). In our study, stress hyperglycemia and subsequent dramatic glucose fluctuations collectively contributed to poor prognosis in patients without diabetes. Some critically ill nondiabetic patients may lose their capacity to maintain glucose homeostasis [26]. Therefore, they may suffer a “double hit” from stress hyperglycemia and high GV. In addition, high SHR levels but low GV in diabetic patients may represent sustained severe stress hyperglycemia and prolonged sympathetic nervous system hyperactivity [44]. Compared with nondiabetic patients, patients with diabetes developed a tolerance to glucose fluctuations during their durations of diabetes. They may be more adversely affected by sustained severe hyperglycemia [44] but less affected by acute glucose excursion [39, 40]. Several other studies based on the ICU population support the validity of our findings [39, 40, 45]. They also noticed that high GV was only associated with poor outcomes in patients without diabetes, whereas it even tended to be a protective factor in patients with diabetes [39, 40, 45]. However, these explanations are speculative due to the lack of data on hormone levels reflecting the hyperactivation of the sympathetic nervous system and the lack of data on markers of inflammation or oxidative stress. Additional studies are required to confirm these hypotheses.

Based on the current evidence, it is worth noting that the transient exposure to stress hyperglycemia and high GV also contribute to long-term adverse cardiovascular outcomes due to the presence of metabolic memory phenomenon. Furthermore, this relationship correlated with the intensity and duration of the exposure [46]. Early detection of stress hyperglycemia and high GV and timely intervention are helpful for improving prognosis. However, after a certain period of poor glycemic control, it was not possible to completely reverse the adverse effects even if good glycemic control was subsequently achieved [46]. Therefore, the existence of the metabolic memory phenomenon encourages us to detect these high-risk factors at an early stage and to correct them with an early reasonable glycemic control regimen to improve the prognosis.

The effects on GV should be considered in selecting antidiabetic agents. Luo et al. found that SGLT-2 inhibitors can decrease GV and improve the function of islet beta-cells in patients with type 2 diabetes treated with insulin in combination with other antidiabetic drugs [25]. In addition, recent studies have also demonstrated that GLP-1 inhibitors contributed to reducing GV [47]. However, all patients in our study were recruited from an ICU. The majority of patients in our study used insulin to control blood glucose levels and the proportion of patients prescribed with other antidiabetic drugs was very low. Therefore, we were unable to further analyze the effects of other antidiabetic drugs on GV and prognosis in the present study.

In summary, the results of the present study may have implications for the clinical practice of glucose management in CAD patients admitted to the ICU. Optimal glycemic management contributes to reducing inflammatory burden, oxidative stress, and infarct size, thereby improving cardiac remodeling in patients with CAD [48, 49]. For some severely ill nondiabetic patients with imbalanced glucose homeostasis, physicians need to monitor their glucose levels closely and decrease their glucose levels smoothly to reduce glucose fluctuations. For diabetic patients, the blood glucose levels should be decreased to a relatively normal range under the premise of avoiding hypoglycemia to prevent them from the potential toxic effects of persistent severe stress hyperglycemia. Therefore, the combined evaluation of SHR and GV may help to guide individualized blood glucose management and improve patient prognosis in the future.

### Strengths and limitations

The primary strength of this study included that our analysis was based on a large, public MIMIC-IV database and the completeness of 1-year follow-up data. However, there are also some limitations in the present study. First, the external validity of our findings is limited due to the retrospective and single-center nature of the present study. Further prospective multicenter studies are required to corroborate our findings. Second, selection bias could be present as we only included patients with available HbA1c data and patients with more than 3 blood glucose measurements. In addition, since the blood glucose data for the present study were extracted from the MIMIC-IV database, the timing of daily blood glucose measurement was not fixed and the number of blood glucose measurements also varied among individuals, which might also introduce bias. However, in our sensitivity analysis where GV was calculated using only blood glucose measurements within 72 h after ICU admission, our main results remained unchanged, indicating the robustness of our results (Supplementary Table 3). Third, given the lack of data on hormone levels representing sympathetic nervous system overactivity and the lack of data on inflammation or oxidative stress markers, the potential mechanisms for the associations of SHR and GV with mortality are speculative. Fourth, fluctuations in blood glucose may be influenced by endogenous and exogenous factors, including the seriousness of the condition in critically ill patients, food intake, nutritional support, insulin dose variations, and the timing of insulin administration. The sensitivity analysis that was further adjusted for the SOFA score demonstrated the robustness of our conclusion (results of model 4 and model 5 in Table 3). However, other factors (food intake, nutritional support, insulin dose variations, and the timing of insulin administration) were not considered in our analysis. Finally, as with the limitations of all observational studies, a cause-and-effect relationship cannot be established from this analysis. In addition, the possibility of residual confounding cannot be completely ruled out despite accounting for many potential confounders.

## Conclusions

Simultaneous assessment of SHR and GV has the potential to improve risk stratification and guide individualized blood glucose management, thereby improving the prognosis of patients.

### Electronic supplementary material

Below is the link to the electronic supplementary material.

Supplementary Material 1

Supplementary Material 2

Supplementary Material 3

## Data Availability

The data of the present study were obtained from the MIMIC-IV database (version 2.2). The availability of these data is restricted, and a license is needed. However, data are available from the author Haoming He (hmhe7411@126.com) upon reasonable request and need to be approved by the Medical Information Mart for Intensive Care Institute.

## Abbreviations

ACEIs/ARBs: Angiotensin-converting enzyme inhibitors/angiotensin receptor blockers
AMI: Acute myocardial infarction
CAD: Coronary artery disease
CI: Confidence interval
eGFR: Estimated glomerular filtration rate
GV: Glycemic variability
HbA1c: Glycated hemoglobin A1c
HR: Hazard ratio
ICU: Intensive care unit
ICD: International Classification of Diseases
MIMIC-IV: Medical Information Mart for Intensive Care-IV
OR: Odds ratio
SHR: Stress hyperglycemia ratio
SOFA: Sequential Organ Failure Assessment
