# Supplementary material for: Simultaneous assessment of stress hyperglycemia ratio and glycemic variability to predict mortality in patients with coronary artery disease: a retrospective cohort study from the MIMIC-IV database
Source: Cardiovasc Diabetol. 2024 Feb 9;23:61. doi: 10.1186/s12933-024-02146-w (PMC10858529; doi:10.1186/s12933-024-02146-w)
Supplement: Supplementary file 1 — Supplementary Material 1 [file 12933_2024_2146_MOESM1_ESM.docx]

**Supplementary Table 1**. Univariate analysis: risk factors of in-hospital and 1-year mortality.

| **Variable** | **Logistic analysis for in-hospital mortality** | | **Cox analysis for 1-year mortality** | |
| --- | --- | --- | --- | --- |
|  | **OR (95% CI)** | ***p* value** | **HR (95% CI)** | ***p* value** |
| SHR | 2.288 (1.835-2.854) | <0.001 | 1.639 (1.457-1.845) | <0.001 |
| GV | 1.021 (1.012-1.031) | <0.001 | 1.017 (1.012-1.023) | <0.001 |
| Age | 1.025 (1.009-1.042) | 0.002 | 1.045 (1.035-1.055) | <0.001 |
| Female | 1.212 (0.844-1.741) | 0.297 | 1.553 (1.272-1.896) | <0.001 |
| Body mass index | 0.982 (0.950-1.016) | 0.296 | 0.981 (0.962-1.001) | 0.062 |
| Systolic blood pressure | 0.996 (0.988-1.004) | 0.346 | 1.004 (0.999-1.008) | 0.093 |
| Diastolic blood pressure | 1.010 (1.000-1.020) | 0.053 | 1.010 (1.005-1.016) | <0.001 |
| Heart rate | 1.024 (1.015-1.034) | <0.001 | 1.019 (1.013-1.024) | <0.001 |
| AMI | 5.936 (3.636-9.692) | <0.001 | 2.919 (2.329-3.659) | <0.001 |
| History of MI | 0.750 (0.452-1.243) | 0.264 | 0.890 (0.678-1.169) | 0.402 |
| Hypertension | 1.259 (0.790-2.006) | 0.332 | 1.504 (1.134-1.995) | 0.005 |
| Diabetes | 0.952 (0.675-1.342) | 0.778 | 1.256 (1.033-1.527) | 0.022 |
| Chronic heart failure | 3.016 (2.099-4.333) | <0.001 | 2.853 (2.325-3.499) | <0.001 |
| Peripheral vascular disease | 1.517 (1.004-2.293) | 0.048 | 1.462 (1.154-1.851) | 0.002 |
| Cerebrovascular disease | 2.555 (1.755-3.719) | <0.001 | 2.252 (1.812-2.799) | <0.001 |
| Antiplatelets | 1.046 (0.637-1.719) | 0.859 | 0.762 (0.591-0.983) | 0.037 |
| Statins | 0.784 (0.534-1.152) | 0.215 | 0.846 (0.677-1.057) | 0.141 |
| ACEIs/ARBs | 0.328 (0.190-0.564) | <0.001 | 0.772 (0.611-0.976) | 0.031 |
| Beta-blockers | 1.078 (0.764-1.521) | 0.670 | 1.054 (0.867-1.282) | 0.598 |
| Vasoactive drugs | 3.051 (1.904-4.887) | <0.001 | 0.942 (0.769-1.155) | 0.567 |
| Insulin | 1.776 (1.220-2.586) | 0.003 | 0.938 (0.770-1.142) | 0.523 |
| Other antidiabetic drugs | 0.441 (0.161-1.207) | 0.111 | 0.447 (0.252-0.794) | 0.006 |
| Renal replacement therapy | 8.180 (5.490-12.187) | <0.001 | 4.325 (3.381-5.532) | <0.001 |
| Ventilation | 2.404 (1.396-4.141) | 0.002 | 0.701 (0.565-0.870) | 0.001 |
| eGFR | 0.975 (0.969-0.981) | <0.001 | 0.980 (0.976-0.983) | <0.001 |

Abbreviations: ACEIs/ARBs, angiotensin-converting enzyme inhibitors/angiotensin receptor blockers; AMI, acute myocardial infarction; CI, confidence interval; eGFR, estimated glomerular filtration rate; GV, glycemic variability; HR, hazard ratio; MI, myocardial infarction; OR, odds ratio; SHR, stress hyperglycemia ratio.
