# Supplementary material for: Simultaneous assessment of stress hyperglycemia ratio and glycemic variability to predict mortality in patients with coronary artery disease: a retrospective cohort study from the MIMIC-IV database
Source: Cardiovasc Diabetol. 2024 Feb 9;23:61. doi: 10.1186/s12933-024-02146-w (PMC10858529; doi:10.1186/s12933-024-02146-w)
Supplement: Supplementary file 2 — Supplementary Material 2 [file 12933_2024_2146_MOESM2_ESM.docx]

**Supplementary Table 2.** Sensitivity analysis: the association of the combination of SHR and GV with mortality in patients with or without diabetes (using a forward stepwise variable selection procedure to build models).

|  | **Group** | | | |
| --- | --- | --- | --- | --- |
|  | **Low SHR and low GV**  **(SHR <1.16 and GV <27.3)** | **High SHR and low GV**  **(SHR >1.16 and GV <27.3)** | **Low SHR and high GV**  **(SHR <1.16 and GV >27.3)** | **High SHR and high GV**  **(SHR >1.16 and GV >27.3)** |
| **In-hospital mortality** | | | | |
| **Overall** | | | | |
| Unadjusted | 1.000 | 3.520  (2.163-5.731)^‡^ | 1.734  (0.975-3.081) | 5.984  (3.743-9.567)^‡^ |
| Model 1 | 1.000 | 4.664 (2.656-8.190)^‡^ | 1.553 (0.770-3.131) | 8.416 (4.862-14.568)^‡^ |
| Model 2 | 1.000 | 3.852 (2.081-7.132)^‡^ | 1.460 (0.692-3.080) | 5.730 (3.106-10.568)^‡^ |
| **Patients without diabetes** | | | | |
| Unadjusted | 1.000 | 3.956  (2.039-7.678)^‡^ | 3.040  (1.155-7.999)^*^ | 14.050  (7.343-26.884)^‡^ |
| Model 1 | 1.000 | 5.508 (2.524-12.024)^‡^ | 4.098 (1.447-11.604)^†^ | 20.024 (9.313-43.052)^‡^ |
| Model 2 | 1.000 | 4.518 (1.922-10.618)^‡^ | 3.564 (1.159-10.961)^*^ | 12.211 (5.168-28.851)^‡^ |
| **Patients with diabetes** | | | | |
| Unadjusted | 1.000 | 3.166  (1.531-6.551)^†^ | 1.121  (0.535-2.351) | 2.512  (1.254-5.032)^†^ |
| Model 1 | 1.000 | 4.111 (1.791-9.433)^‡^ | 0.757 (0.290-1.979) | 3.307 (1.463-7.475)^†^ |
| Model 2 | 1.000 | 4.277 (1.575-11.615)^†^ | 0.941 (0.329-2.689) | 3.477 (1.320-9.158)^*^ |
| **1-year mortality^a^** | | | | |
| **Overall** | | | | |
| Unadjusted | 1.000 | 2.243 (1.702-2.957)^‡^ | 1.680 (1.215-2.324)^†^ | 3.336 (2.372-4.692)^‡^ |
| Model 1 | 1.000 | 3.024 (2.184-4.187)^‡^ | 2.137 (1.454-3.140)^‡^ | 4.667 (3.088-7.051)^‡^ |
| Model 3 | 1.000 | 2.418 (1.740-3.361)^‡^ | 1.998 (1.350-2.956)^‡^ | 3.048 (1.999-4.649)^‡^ |
| **Patients without diabetes** | | | | |
| Unadjusted | 1.000 | 2.193 (1.492-3.222)^‡^ | 3.404 (2.025-5.722)^‡^ | 5.998 (3.704-9.714)^‡^ |
| Model 1 | 1.000 | 3.272 (2.066-5.183)^‡^ | 4.770 (2.614-8.705)^‡^ | 9.336 (5.248-16.605)^‡^ |
| Model 3 | 1.000 | 2.678 (1.682-4.263)^‡^ | 4.050 (2.195-7.472)^‡^ | 6.117 (3.401-11.001)^‡^ |
| **Patients with diabetes** | | | | |
| Unadjusted | 1.000 | 2.589 (1.727-3.880)^‡^ | 1.059 (0.685-1.636) | 2.003 (1.223-3.279)^†^ |
| Model 1 | 1.000 | 3.170 (1.975-5.088)^‡^ | 1.158 (0.688-1.949) | 2.391 (1.304-4.382)^†^ |
| Model 3 | 1.000 | 2.289 (1.410-3.717)^‡^ | 1.256 (0.740-2.130) | 1.755 (0.942-3.270) |

Abbreviations: GV, glycemic variability; SHR, stress hyperglycemia ratio.

^a^The assumption of proportional hazards was not met. Therefore, HRs were calculated using Cox regression analysis with time-dependent covariates.

Model 1: adjusted for age, female, and body mass index.

Model 2: adjusted for Model 1 plus acute myocardial infarction, chronic heart failure, cerebrovascular disease, angiotensin-converting enzyme inhibitors/angiotensin receptor blockers, vasoactive drugs, insulin, other antidiabetic drugs, renal replacement therapy, ventilation, and estimated glomerular filtration rate.

Model 3: adjusted for Model 1 plus acute myocardial infarction, chronic heart failure, cerebrovascular disease, insulin, other antidiabetic drugs, renal replacement therapy, and estimated glomerular filtration rate.

^*^P<0.05, ^†^P<0.01, ^‡^P<0.001.
