# Supplementary material for: Simultaneous assessment of stress hyperglycemia ratio and glycemic variability to predict mortality in patients with coronary artery disease: a retrospective cohort study from the MIMIC-IV database
Source: Cardiovasc Diabetol. 2024 Feb 9;23:61. doi: 10.1186/s12933-024-02146-w (PMC10858529; doi:10.1186/s12933-024-02146-w)
Supplement: Supplementary file 3 — Supplementary Material 3 [file 12933_2024_2146_MOESM3_ESM.docx]

**Supplementary Table 3.** Sensitivity analysis: the association of the combination of SHR and GV with mortality in patients with or without diabetes (GV was calculated by using blood glucose measurements within 72 hours after ICU admission).

|  | **Group** | | | |
| --- | --- | --- | --- | --- |
|  | **Low SHR and low GV**  **(SHR <1.16 and GV <27.3)** | **High SHR and low GV**  **(SHR >1.16 and GV <27.3)** | **Low SHR and high GV**  **(SHR <1.16 and GV >27.3)** | **High SHR and high GV**  **(SHR >1.16 and GV >27.3)** |
| **In-hospital mortality** | | | | |
| **Overall** | | | | |
| Unadjusted | 1.000 | 3.113 (1.971-4.916)^‡^ | 1.028 (0.540-1.955) | 4.794 (3.037-7.568)^‡^ |
| Model 1 | 1.000 | 4.233 (2.522-7.105)^‡^ | 0.854 (0.383-1.902) | 6.282 (3.703-10.658)^‡^ |
| Model 2 | 1.000 | 3.368 (1.887-6.009)^‡^ | 0.779 (0.336-1.803) | 4.000 (2.201-7.270)^‡^ |
| **Patients without diabetes** | | | | |
| Unadjusted | 1.000 | 3.657 (1.978-6.761)^‡^ | 1.380 (0.399-4.768) | 10.268 (5.429-19.418)^‡^ |
| Model 1 | 1.000 | 5.091 (2.536-10.219)^‡^ | 1.854 (0.517-6.653) | 13.228 (6.370-27.467)^‡^ |
| Model 2 | 1.000 | 4.293 (1.983-9.293)^‡^ | 1.569 (0.408-6.032) | 7.727 (3.366-17.737)^‡^ |
| **Patients with diabetes** | | | | |
| Unadjusted | 1.000 | 2.608 (1.300-5.231)^†^ | 0.750 (0.342-1.644) | 2.309 (1.189-4.484)^*^ |
| Model 1 | 1.000 | 3.456 (1.559-7.663)^†^ | 0.510 (0.180-1.448) | 3.033 (1.387-6.635)^†^ |
| Model 2 | 1.000 | 3.527 (1.349-9.221)^*^ | 0.607 (0.199-1.852) | 3.222 (1.290-8.046)^*^ |
| **1-year mortality^a^** | | | | |
| **Overall** | | | | |
| Unadjusted | 1.000 | 2.171 (1.659-2.842)^‡^ | 1.424 (1.012-2.002)^*^ | 3.227 (2.293-4.543)^‡^ |
| Model 1 | 1.000 | 2.992 (2.186-4.096)^‡^ | 1.751 (1.170-2.621)^†^ | 4.219 (2.792-6.373)^‡^ |
| Model 3 | 1.000 | 2.413 (1.756-3.316)^‡^ | 1.674 (1.114-2.517)^*^ | 2.851 (1.873-4.340)^‡^ |
| **Patients without diabetes** | | | | |
| Unadjusted | 1.000 | 2.353 (1.612-3.434)^‡^ | 2.416 (1.312-4.446)^†^ | 5.549 (3.356-9.174)^‡^ |
| Model 1 | 1.000 | 3.886 (2.455-6.150)^‡^ | 3.538 (1.729-7.240)^‡^ | 8.607 (4.678-15.835)^‡^ |
| Model 3 | 1.000 | 3.199 (2.010-5.092)^‡^ | 2.962 (1.438-6.102)^†^ | 5.512 (2.967-10.242)^‡^ |
| **Patients with diabetes** | | | | |
| Unadjusted | 1.000 | 2.342 (1.568-3.497)^‡^ | 1.012 (0.651-1.574) | 2.134 (1.313-3.468)^†^ |
| Model 1 | 1.000 | 2.885 (1.806-4.607)^‡^ | 1.107 (0.656-1.867) | 2.488 (1.376-4.501)^†^ |
| Model 3 | 1.000 | 2.129 (1.315-3.445)^†^ | 1.279 (0.754-2.167) | 1.986 (1.083-3.640)^*^ |

Abbreviations: ICU, intensive care unit; GV, glycemic variability; SHR, stress hyperglycemia ratio.

^a^The assumption of proportional hazards was not met. Therefore, HRs were calculated using Cox regression analysis with time-dependent covariates.

Model 1: adjusted for age, female, and body mass index.

Model 2: adjusted for Model 1 plus acute myocardial infarction, chronic heart failure, cerebrovascular disease, angiotensin-converting enzyme inhibitors/angiotensin receptor blockers, vasoactive drugs, insulin, other antidiabetic drugs, renal replacement therapy, ventilation, and estimated glomerular filtration rate.

Model 3: adjusted for Model 1 plus acute myocardial infarction, chronic heart failure, cerebrovascular disease, insulin, other antidiabetic drugs, renal replacement therapy, and estimated glomerular filtration rate.

^*^P<0.05, ^†^P<0.01, ^‡^P<0.001.
